# The nutritional value, bioactive availability and functional properties of garlic and its related products during processing

**DOI:** 10.3389/fnut.2023.1142784

**Published:** 2023-07-25

**Authors:** Piyachat Sunanta, Vassilis Kontogiorgos, Tanachai Pankasemsuk, Kittisak Jantanasakulwong, Pornchai Rachtanapun, Phisit Seesuriyachan, Sarana Rose Sommano

**Affiliations:** ^1^Multidisciplinary Research Institute, Chiang Mai University, Chiang Mai, Thailand; ^2^Plant Bioactive Compound Laboratory (BAC), Faculty of Agriculture, Chiang Mai University, Chiang Mai, Thailand; ^3^Food, Nutrition and Health, Faculty of Land and Food Systems, The University of British Columbia, Vancouver, BC, Canada; ^4^Department of Plant and Soil Science, Faculty of Agriculture, Chiang Mai University, Chiang Mai, Thailand; ^5^School of Agro-Industry, Faculty of Agro-Industry, Chiang Mai University, Chiang Mai, Thailand; ^6^Center of Excellence in Agro Bio-Circular-Green Industry (Agro BCG), Agro-Industry, Chiang Mai University, Chiang Mai, Thailand

**Keywords:** physical properties, chemical properties, chemical alteration, functional food, health beneficial

## Abstract

Garlic, a common culinary spice, is cultivated and used around the globe. Consumption of garlic and its supplements reduces the risk of diabetes and cardiovascular disease and boosts the immune system with antibacterial, antifungal, anti-aging, and anti-cancer properties. Diallyl sulfide, diallyl disulfide, triallyl trisulfide, phenolics, flavonoids, and others are the most commercially recognized active ingredients in garlic and its products. In recent years, global demand for medicinal or functional garlic has surged, introducing several products such as garlic oil, aged garlic, black garlic, and inulin into the market. Garlic processing has been demonstrated to directly impact the availability of bioactive ingredients and the functionality of products. Depending on the anticipated functional qualities, it is also recommended that one or a combination of processing techniques be deemed desirable over the others. This work describes the steps involved in processing fresh garlic into products and their physicochemical alterations during processing. Their nutritional, phytochemical, and functional properties are also reviewed. Considering the high demand for functional food, this review has been compiled to provide guidance for food producers on the industrial utilization and suitability of garlic for new product development.

## 1. Introduction

Garlic is a multipurpose plant widely cultivated and consumed worldwide with a long history in culinary, medical, and trading (1, 2). It is commonly used as a spice and vegetable for enhancing flavor and comes in various forms, including bulb and chive, with some species used as ornamental and landscape plants (3). In folk medicine, garlic has been used to treat ailments such as typhus, dysentery, cholera, and influenza since ancient times (4). Consumption of garlic and its supplements is proven to lower the risk of diabetes and cardiovascular disease, defend against infections by activating the immune system, and have antibacterial, antifungal, anti-aging, and anti-cancer properties (5–7). Over 300 garlic varieties have been recognized, collectively categorized into two major types: soft-neck garlic (*Allium sativum* var. *sativum*) and hard-neck garlic (*A. sativum* var. ophioscorodon). Taxonomically, the hard-neck type produces flowering pods, which are not apparent in the soft-neck garlic species. Even though garlic has demonstrated significant genetic variation in terms of morphology, the genotype has almost no effect on nutritional and functional properties (8–10). In 2020, global garlic production was over 28 million tonnes with a value of up to US $17,000 million, and China has been by far the world’s largest producer and exporter of garlic (11). In recent years, global demands for garlic for medicinal or functional foods have increased, with a wide variety of products available in the markets. The processing involves initial processing such as cleaning, cutting, drying and food preservation, which generate over 3.7 million tonnes of garlic by-products annually (11). Garlic contains approximately 200 chemical compounds, including sulfur-containing compounds, volatile oils, enzymes, carbohydrates, minerals, amino acids, and vitamins (12, 13). S-allyl-L-cysteine sulfoxides or alliin, an odorless chemical substance, are the main bioactive ingredient found in undamaged fresh garlic and other S-alkyl-L-cysteine sulfoxides (14). The products include chopped, dehydrated, fried, aged garlic, and garlic oil (15–17). The processing steps have been confirmed to impact the final bioactive potency directly, and slight differences in the level of active ingredients may also cause profound variations in the bioavailability and final bioactivity of the final products (18–20). It is also advised that a combination of processing techniques should be preferred, depending on the expected functional properties (19). On this basis, the present review elucidates the processes involved in producing different garlic products and describes the related biochemical mechanisms involve in the alterations of their chemical and bioactive composition.

## 2. Bioactive constituents and functional ingredients in garlic

Garlic has garnered considerable attention in the functional food industry owing to its exceptional bioactive constituents and functional ingredients that contribute to its medicinal properties. Encompasses a diverse range of sulfur-containing compounds, such as allicin, diallyl sulfide, diallyl disulfide, and diallyl trisulphide, which have been the subject of extensive scientific investigation due to their notable therapeutic effects (21, 22). Vitamins (e.g., vitamin C, B vitamins) and minerals (e.g., selenium) contribute to garlic’s overall nutritional value and health benefits (23, 24). The investigation of the bioactive compounds and functional ingredients of garlic establishes a solid foundation for comprehending its potential therapeutic applications (25). This emphasis further understanding the intrinsic value of garlic as superfood with the potential in natural medicine and pharmacology.

### 2.1. Allicin

Allicin, is known for its antimicrobial, anti-inflammatory, and antioxidant properties; it is formed enzymatically from the precursor alliin upon garlic tissue injury (26, 27). It is responsible for the distinctive odor of garlic, contributes to several of its therapeutic effects, and is recognized as the bioactive compound that has attracted considerable scientific attention due to its powerful bioactivity (28, 29).The enzymatic interaction between the enzyme alliinase and a sulfur-containing compound (alliin) is responsible for the generation of allicin, which occurs upon disruption of garlic (30). Following ingestion, allicin is swiftly absorbed from the digestive system into the bloodstream, traverses cell membranes, and gains access to various tissues (31). As a result of its inherent instabilities, allicin undergoes rapid conversion into diallyl sulfide, diallyl disulfide, and diallyl trisulphide (32, 33). The metabolites of allicin are primarily eliminated from the body through urine and exhalation. The rate of allicin elimination varies depending on various factors, including individual variations and the specific dosage form administered. Allicin displays plenty of advantageous properties, such as antimicrobial activity that inhibits the growth of bacteria, viruses, and fungi through multiple mechanisms. In addition, it acts as an antioxidant that neutralizes free radicals (30, 34). In addition, allicin shows anti-inflammatory effects by inhibiting mediators of inflammation and enzymes, showing that it may be beneficial in inflammatory conditions (30, 35). This compound can enhance blood flow and cardiovascular health by reducing blood pressure, inhibiting platelet aggregation, and vasodilating (2).

### 2.2. Organosulfur compounds

Garlic contains organosulfur compounds, including S-allylcysteine (SAC) and ajoene. SAC is a bioactive compound generated through the breakdown of allicin during garlic aging or processing (36). SAC is a compound that is soluble in water and may have numerous health benefits (37). Due to its solubility in water, SAC is readily absorbed from the intestines after consumption and then diffuses throughout the entire bloodstream. It can penetrate tissues, including those of the liver, kidneys, heart, and brain (38). The studies indicate that SAC may be degraded by cysteine dioxygenase and glutamyl transpeptidase, leading to the formation of cysteine and other metabolites that may contribute to its biological activities (39, 40).

### 2.3. Another sulfur compounds

Garlic contains an abundance of sulfur-containing compounds or oil soluble compounds, including diallyl sulfide (DAS), diallyl disulfide (DADS) and diallyl trisulfide (DATS) (41, 42). These compounds are products of allicin which normally found in garlic oil (22). Scientific research has substantiated that these sulfur compounds exhibit an extensive array of biological activities, notably including antioxidant properties antimicrobial, anti-inflammatory, anticancer, and cardioprotective properties (43, 44).

### 2.4. Antioxidant compounds

Several antioxidant compounds that occur in garlic contribute to its overall antioxidant activity. It serves an important purpose in maintaining cellular health and has been connected to various health benefits. Apart from the sulfur compounds, garlic contains phenolic and flavonoid compounds, which greatly contribute to its antioxidant capacity (45, 46). Garlic is abundant in phenolic compounds such as caffeic acid, p-coumaric acid, ferulic acid, and their respective derivatives (47). However, flavonoids are the most common plant antioxidant compounds, alongside quercetin, kaempferol, and apigenin, typically found in garlic (47, 48). Significantly, the levels and varieties of antioxidant compounds in garlic may differ depending on factors such as garlic variety, cultivation conditions, and processing methods (15, 49). In addition, the bioavailability and potential beneficial interactions of those compounds throughout the human body are still under research.

## 3. Pre-harvesting

Some cultivation factors may influence the quality of raw garlic, particularly environmental factors such as climatic conditions, photoperiod, and soil quality (50, 51). Garlic grows most effectively in soils that are well-drained, rich in organic matter and nutrients, and mildly acidic to neutral in pH (52). Temperature and precipitation patterns can also affect the growth and quality of garlic (53, 54). Garlic is also a photoperiodic plant, which means its growth is influenced by the period of daylight. However, the optimal photoperiod for garlic differs depending on the variety (55). Proper photoperiod exposure helps regulate bulb formation and development. In the research of Atif et al. (55), which examined the effect of photoperiod on the concentration of garlic’s functional constituents, 14 h of photoperiod produced the maximum concentration of chemical compounds in the bulb.

Importantly, cultivation practices such as water management, fertilization management, and disease and insect control play a significant role in crop quality (56–58). Garlic bulbs are susceptible to fungal diseases such as white rot and purple blotch (59). Effective disease and pest control measures, such as crop rotation, sanitation, and the use of fungicides and insecticides, assist in minimizing yield losses and preserving quality (56). Along with the time of harvest, the timing of garlic gathering may impact its quality. Early harvesting may result in immature cloves with a mild taste and a shorter shelf life. In contrast, delayed harvesting can result in overripe cloves with a stronger flavor and a higher tendency to sprout (60).

## 4. Post-harvesting

**Figure 1:**
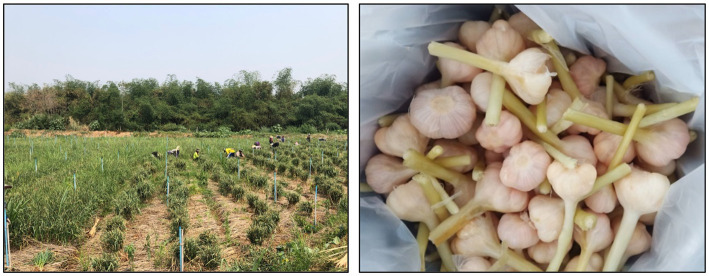
**(A)** Garlic harvesting in Thailand; **(B)** Cleaned fresh harvesting garlic for garlic pickle processing.

The garlic bulb consists of several layers of storage leaves attached to the basal plate (61, 62). As mentioned, garlic is morphologically classified into two sub-species, either hard- or soft-neck garlic, according to the development of flower stalks, hardiness, and the pattern of clove formation. The hard-neck has elongated flower stalks with a flower at the top of the stalk, whereas soft-neck garlic does not produce a seed stalk (63, 64). Hard-neck garlic takes around 6 months to grow and prefers a cool climate for storing nutrients. Consequently, the bulb and clove are typically larger than soft-neck garlic, composed of four to 12 small cloves surrounding the flower stalk. They can be stored for several months after harvesting (64). The cultivation of soft-neck garlic is shorter (3–4 months) in a tropical region, and basically, the bulb is composed of small 10–40 cloves arranged in multiple layers around the basal plate (9, 64). It is also found that the soft-neck types have a longer storage life than the hard-neck cultivars, which can be stored for up to 1 year (65). However, according to Sunanta et al. (9), the antioxidant properties of both subspecies were not significantly different. Cloves mainly propagate garlic due to its sexual sterility, results in has a lower genetic improvement efficiency (66, 67). When the top of the leaf turns brown at the harvesting stage, the plant is plucked from the ground, and the roots are still attached (68) (Figure 1A). The entire bulb of freshly harvested garlic is top-cut and cleaned for garlic pickles before being pickled in vinegar, brine, or another solution (Figure 1B). However, fresh garlic consumption requires a curing process to enhance its quality and shelf life.

### 4.1. Curing

**Figure 2:**
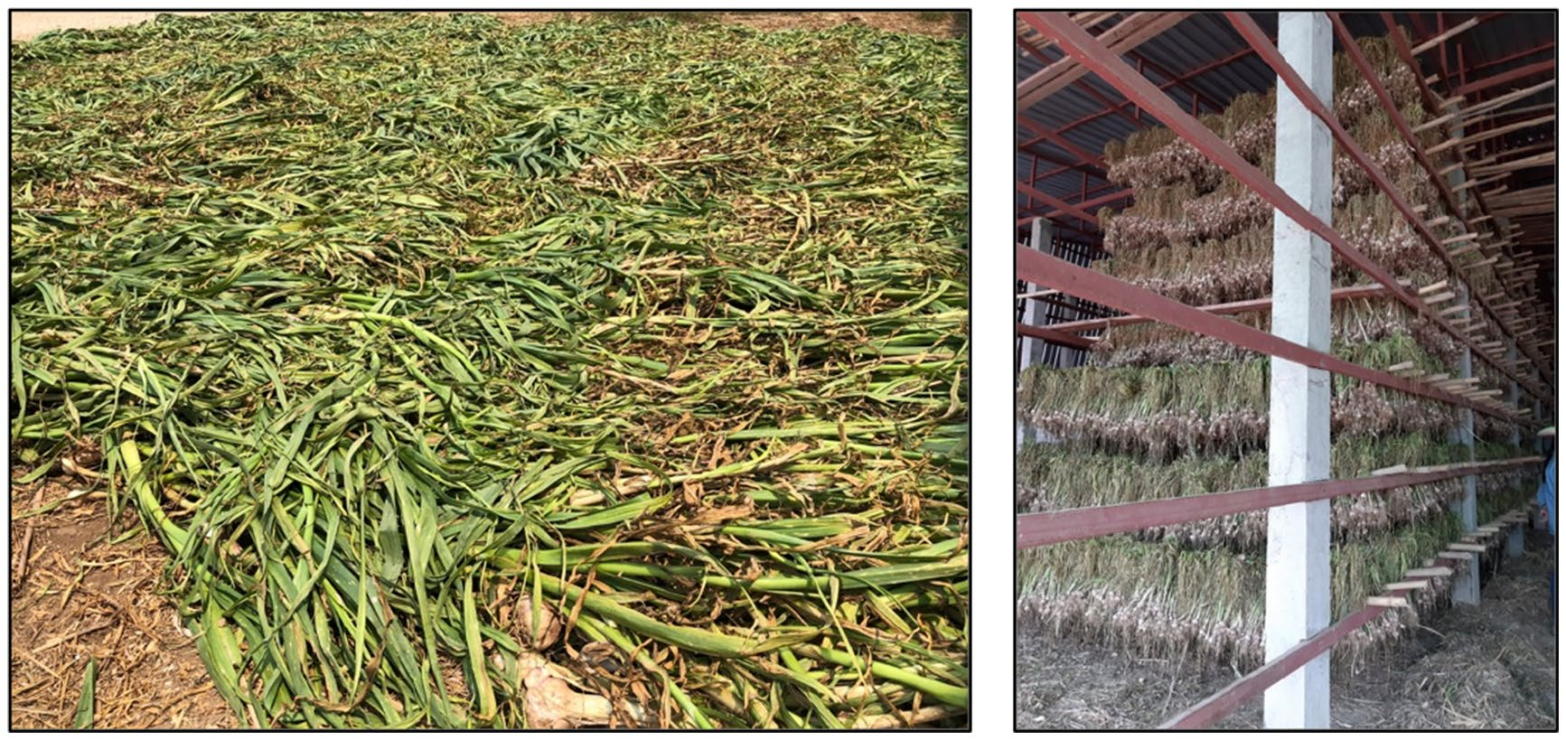
Natural curing **(A)** Sun drying; freshly harvested garlic is spread on the field and covered with the leaves; **(B)** Shade drying; harvested garlic is bunched up and hung in the shade.

Curing is a vital post-harvest step that increases garlic’s marketability and shelf life by removing excess moisture, resulting in a dried bulb with an attractive skin color (69, 70). The neck and skin are dehydrated, forming a protective barrier around the bulb and preventing fungal and bacterial infection during storage (71). According to Medina and Garcia (18), the temperature and relative humidity of the curing process affect the flavor of garlic. The curing process can be applied either naturally or using artificial heat. In hot climates, freshly harvested garlic is typically dried by spreading it on the ground and covering it with its leaves for 1–2 weeks to prevent sunburn (72) (Figure 2A). In another technique, natural curing is accomplished by bunching the entire garlic plant and hanging it in the shade (Figure 2B) until the outer skin shrinks and forms a thin layer over the garlic bulbs (69).

However, due to the unpredictable nature of the weather, natural curing may have a detrimental effect on the quality of dry skin and shorten the storage life because of sprouting and bulb rotting (73). Artificial curing can be achieved by circulating hot air through the product. Although temperatures between 25 and 35°C with 65–80% relative humidity have been recommended for garlic curing, temperatures greater than 35°C should be avoided due to the significant risk of bacterial infection and the development of several diseases (74). Curing has a substantial impact on the physical and chemical properties of garlic. Bayat et al. (75) reported that after 7 days of natural curing, the moisture content of the cloves was reduced to 32%, but their firmness remained stable. In contrast, artificial curing at 35, 45, and 55°C took 7, 4, and 1 day(s), respectively. However, the pyruvate concentration decreases when garlic bulbs are cured at high temperatures (>35°C). As a result of the decrease in alliinase activity, taste precursors are degraded (76). To conclude, curing at various temperatures and using various procedures significantly impacts the bulbs’ quality and storage stability. After the curing process is complete, the garlic will be graded and sorted prior to being stored and sold, with the primary aim of expanding its value and minimizing storage losses. Table 1 illustrates the various curing processes used in garlic. According to the data in the table, natural curing temperature and relative humidity are uncontrollable and depend on the weather in each country. Furthermore, the curing time was uncertain because it was influenced by the curing condition. This method of curing is both inexpensive and simple. To manage the conditions of the curing process, artificial curing was developed. This procedure was carried out in a room with a temperature, relative humidity, and airflow that could be controlled.

**Table 1:** The physiochemical characteristics of garlic and their curing condition.

Curing processes	Curing condition		Physiochemical properties			Reference
	Temperature	Duration	Moisture content	Pyruvate content	Firmness	
Sun drying	> 35°C	1-4 weeks	N/R	↓	N/R	Opara (72)
Shade drying	< 35°C	>3 weeks	↓ 31.33%	↑ 9.62%	↑ 0.52%	Bayat et al. (75)
Oven heating	35°C	1 week	↓ 31.33%	↓ 9.62%	↓ 6.51%	Bayat et al. (75)	45°C	3 days	↓ 31.33%	↓ 2.24%	↓ 5.41%	Bayat et al. (75)	55°C	1 day	↓ 31.33%	↓ 24.20%.	↑ 2.97%	Bayat et al. (75)

Physiochemical properties values were compared to those of fresh garlic. ↓ and ↑ indicate a decrease and an increase, respectively, from freshly harvested garlic.

N/R is not reported.

The duration of garlic curing was dependent on temperature and drying method. After drying, garlic’s moisture content decreased considerably. When the curing temperature was increased, the final clove firmness fell dramatically. However, at 55°C of drying temperature, the outside of garlic cloves became hardened, affecting the firmness value. On the other hand, the pyruvate content was unaffected by the temperature. However, there have been a few studies on garlic curing and the chemical alteration that occurs during the process.

### 4.2. Minimal processing

**Figure 3:**
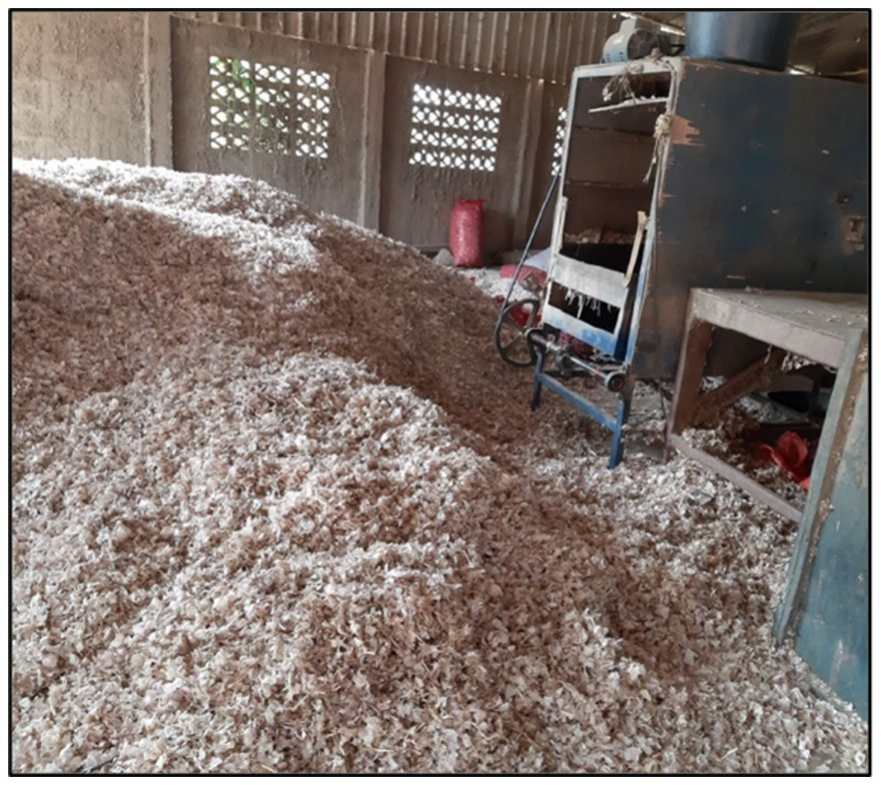
Garlic dry-peeling.

Minimal processing aims for convenient, fresh-tasting products with acceptable nutritional and sensory characteristics. Demand for fresh fruit and vegetables is growing, increasing the amount and variety of products available to consumers, while several factors limit the product’s quality and shelf life (77). The nutritional value, weight, flavor, and color of fresh foods continue to deteriorate internally and externally over time due to morphological and physiological damages, storage conditions, and chemical treatments (77, 78). Consequently, effective processes are essential to maintaining product quality and extending the life of the product. To achieve maximum consumer convenience, washing, peeling, and cutting are frequently used as minimal processes. However, these processes result in numerous physiological and microbiological changes that degrade product characteristics, resulting in a shorter shelf life due to damaged tissue during processing (79). Washing efficiently removes microorganisms, dirt, pesticides, and nutrition leaks from cells (80). To thoroughly clean and sanitize, chemical substances such as chlorine, hydrogen peroxide, and ozone were applied (81). On the other hand, peeling and cutting may increase the chances of contamination. According to Park et al. (79), peeling eliminates the inedible parts of garlic, such as the skin and stalk, which can be separated during dry and wet peeling (Figure 3). Additionally, dry peeling garlic may result in a lower microbial concentration than wet peeling, as it was considered that the microbes on the skin dissolved in water and infected the garlic flesh. Excessive washing, on the other hand, may result in the loss of soluble nutrients. Cutting, slicing, dicing, and shredding reduce the product’s size and the time required for preparation. On the contrary, cutting accumulates fluids on the cut surface, increasing microbial load and enzyme activity and resulting in a shorter storage life (82).

The alliinase enzyme and alliin are localized in different microcompartments in garlic cloves. Alliin can interact with alliinase when the compartments have been disrupted (e.g., sliced, chopped, or crushed) (26, 27). Allicin is a defensive component that gives garlic its taste and smell (28). Allicin decomposes in the stomach after raw garlic is consumed, releasing volatile compounds (e.g., allyl sulfides, disulfides, and other volatiles) associated with the garlic smell (21). Nonetheless, allicin has been reported to have a wide range of pharmacological effects, including antibacterial, antioxidant, anticarcinogenic, and antifungal properties (83, 84). Because allicin binds to the protein of red blood cells and oxidizes it instantly, Freeman and Kodera (85) suggested that the remaining allicin was rapidly eliminated from whole blood, while DAS and allyl mercaptan were generated after consuming raw garlic. It is assumed that if allicin is taken orally, it immediately attaches to the lumen and is retained because of the intense sensation in the mouth after chewing the raw garlic clove. As a result, it does not pass through the digestive system membrane and travels through the bloodstream via the serosa (86). These studies show that allicin does not play a role in any of garlic’s therapeutic benefits inside the body. During the physical damage of raw garlic, γ-glutamyl-S-allyl-L-cysteine was also converted to S-allyl-cysteine (SAC), a water-soluble sulfur compound (86, 87). Liu and Yeh (88) reported that the SAC efficiently lowered cholesterol production by up to 55% and was not cytotoxic. Additionally, the Allium genus has been discovered as a rich source of bioactive components, such as polyphenols, flavonoids, and their derivatives, that have all been associated with health benefits (89).

Although minimally processed garlic is accessible to consumers, the raw material becomes highly perishable because of the damaged tissues generated during processing. This makes it more susceptible to microbiological spoilage, physicochemical degradation, and sensory depletion (90). As a result, minimally processed garlic typically has a significantly shorter shelf life than intact products. Usually, garlic cloves are wrapped with a peel that regulates oxygen, carbon dioxide, and moisture transportation and also helps prevent flavor and aroma loss, but minimally processed garlic eliminates this barrier. Edible surface coating is a simple and safe process for replacing the natural barrier on fresh-cut products and enhancing their quality (91). These coatings protect the products from water loss and contribute to retaining nutrients and reducing microbial deterioration. Additionally, edible coatings would enhance the physical, sensory, and shelf life of the product (92). The list of edible coatings for extending the shelf-life of garlic is shown in Table 2.

**Table 2:** The coating process of fresh garlic clove.

Coating component	Coating process	Physiochemical properties				Reference
		Moisture loss	Respiration rate	Fungi count	Allicin content	
Agar	1% agar solution	↓ 66.67%	↓ 27.27%	↑ 28.57%	N/R	Geraldine et al. (93)	1% agar solution with 0.2% chitosan	↓ 66.67%	↓ 45.45%	↓ 28.57%	N/R	Geraldine et al. (93)	1% agar solution with 0.2% acetic acid	↓ 66.67%	↓ 27.27%	↓ 28.57%	N/R	Geraldine et al. (93)
Alginate	2% sodium alginate	↓ 9.71%	↑ 33.33%	N/R	N/R	Nussinovitch and Hershko (94)	2% sodium alginate with β-sitosterol	↓ 18.86%	↑ 53.33%	N/R	N/R	Nussinovitch and Hershko (94)
Gellan	2% Gellan gum	↑↓	↑ 25.00%	N/R	N/R	Nussinovitch and Hershko (94)	2% Gellan gum with β-sitosterol	↑↓	↑ 112.50%	N/R	N/R	Nussinovitch and Hershko (94)
Protein	20% Zein	↓ 13.23%	N/R	N/R	↑ 17.65%	Torun and Ozdemir (95)	15% milk protein	↓ 7.84%	N/R	N/R	↑ 41.18%	Torun and Ozdemir (95)

Physiochemical properties values were compared to those of uncoated garlic. ↓ and ↑ indicate a decrease and an increase, respectively, from freshly harvested garlic.

N/R is not reported.

Typically, the coating can minimize the moisture loss rate, while the rate of respiration and the rate of microorganism growth depend on the composition. Coating with agar lowered the respiration rate and the development of fungi. On the other hand, other coating components enhanced their respiration rate. However, data on the physiochemical properties of coated garlic during storage is lacking.

### 4.3. Drying

Food dehydration is a preservation technique that removes up to 90% of the moisture from food. Fresh garlic has a high moisture content (more than 75%), which promotes garlic clove sprouting and rotting during storage, resulting in a shorter shelf life (96). Thus, to extend garlic’s shelf life while maintaining its nutritional value, the moisture content of garlic must be reduced. Thermal (hot air drying, infrared drying, microwave drying, vacuum drying) and non-thermal (freeze-drying) drying technologies are common practices in the food industry (97). Drying kinetics, physical qualities (such as color, density, shrinkage, and hardness), and chemical properties (such as allicin content, antioxidant activity, total phenolic content, and volatile components) of garlic were all adversely affected by each drying procedure (98–100). According to Madamba et al. (101), temperature and the thickness of the garlic slice played a significant role in the drying process, although relative humidity and air speed had almost no effect on the drying rate.

The color of dehydrated garlic is determined by the temperature of the drying process, which is primarily based on the Maillard and caramelization reactions. As a result, dehydrated garlic obtained from thermal drying has a dark color (102). Furthermore, due to the rapid evaporation of surface moisture from thermal drying, which generated a pressure difference between the inside and outside of the material, the shrinkage ratio of garlic products was also dependent on the dehydration temperature, causing the surface to be drawn to the center (103). Additionally, the shrinkage ratio and the hardness value of dried garlic have a strong relationship. However, because the enzyme alliinase, which converts alliin to allicin, is inactivated by heat, the volatile chemicals produced by non-thermal drying differed significantly from those produced by thermal drying. Because of its distinctive odor, allicin has long been assumed to be the active compound (86, 104). The other bioactive components (e.g., phenolics and flavonoids) in dehydrated garlic were reduced due to the drying temperature, drying time, and reduced water activity (105, 106). To summarize, drying can increase the shelf life of the product while also reducing microbial activity and, consequently, reducing the transportation expenses (107). Table 3 shows the drying techniques used in the garlic industry. The drying process decreased the total phenolic, pyruvate, and allicin content compared to fresh garlic. Garlic can be ground into a fine powder after drying and sold as garlic powder. Garlic slices dried faster when thinner, but the smaller the garlic slice, the more alliin was reduced in the garlic powder (104). As a result, dried garlic powder typically has 50% less alliin than fresh garlic. Garlic powder contains no allicin, possibly accounting for its instability (110).

**Table 3:** Different drying approaches used in the industry and their analyzed physiochemical properties.

Drying technique	Condition			Physiochemical properties				Reference
	Temperature	Duration	Sample thickness	Moisture content	Pyruvate content	Allicin content	Total phenolic content	
Hot air drying	40°C	30 h	Clove	↓ 96.76%	N/R	N/R	N/R	Sharma and Prasad (108)
	50°C	25 h	Clove	↓ 96.76%	N/R	N/R	N/R	
	60°C	11.5 h	Clove	↓ 96.76%	N/R	N/R	N/R	
	70°C	6.5 h	Clove	↓ 96.76%	N/R	N/R	N/R	
	60°C	6 h	3 mm	↓ 91.83%	N/R	↓ 23.03%	↓ 57.47%	Feng et al. (106)
	60-65°C	6 h	3 mm	↓ 95.50%	↓ 45.84%	N/R	N/R	Cui et al. (109)
Freeze-drying	-95 °C	13.5 h	3 mm	↓ 91.57%	N/R	↓ 15.69%	↓ 52.59%	Feng et al. (106)
	45 °C	1 h	3 mm	↓ 95.30%	↓ 2.22%	N/R	N/R	Cui et al. (109)
Microwave-convective drying (10 W)	40°C	6.91 h	Clove	↓ 96.76%	N/R	N/R	N/R	Sharma and Prasad (108)
	50°C	4 h	Clove	↓ 96.76%	N/R	N/R	N/R	
	60°C	2.83 h	Clove	↓ 96.76%	N/R	N/R	N/R	
	70°C	2.50 h	Clove	↓ 96.76%	N/R	N/R	N/R	
Microwave-convective drying (20 W)	40°C	3.251 h	Clove	↓ 96.76%	N/R	N/R	N/R	Sharma and Prasad (108)
	50°C	2 h	Clove	↓ 96.76%	N/R	N/R	N/R	
	60°C	1.33 h	Clove	↓ 96.76%	N/R	N/R	N/R	
	70°C	1.08 h	Clove	↓ 96.76%	N/R	N/R	N/R	
Microwave-convective drying (40 W)	40°C	2.5 h	Clove	↓ 96.76%	N/R	N/R	N/R	Sharma and Prasad (108)
	50°C	1.5 h	Clove	↓ 96.76%	N/R	N/R	N/R	
	60°C	1.33 h	Clove	↓ 96.76%	N/R	N/R	N/R	
	70°C	1 h	Clove	↓ 96.76%	N/R	N/R	N/R	
Microwave vacuum drying (750W)	N/R	35 min	3 mm	↓ 95.80%	↓ 11.18%	N/R	N/R	Cui et al. (109)
Infrared drying	60 °C	3.8 h	3 mm	↓ 91.66%	N/R	↓ 12.79%	↓ 45.69%	Feng et al. (106)
Pulsed vacuum drying	60 °C	4.8 h	3 mm	↓ 91.39%	N/R	↓ 21.01%	↓ 54.60%	Feng et al. (106)

Physiochemical properties values were compared to those of fresh garlic. ↓ and ↑ indicate a decrease and an increase, respectively, from freshly harvested garlic.

N/R is not reported.

The drying period of garlic varied depending on a number of variables, including methodology, temperature, and the pre-processing method. A high temperature can decrease the drying period, but the physiochemical variations between dried garlic produced using various drying techniques have not been widely reported. After drying, the total phenolic and allicin content decrease dramatically, according to the presented research.

## 5. Garlic products

In recent years, there has been considerable growth in the functional food industry (111). Food processing is commonly used to improve product stability, microbiological stability, and the ability to deactivate enzymes; however, it can also improve food quality and stability by providing chemical, physical, and nutritional improvements (92, 111). Garlic’s market expansion has been limited due to its intense odor, which is not tolerated by many consumers (112). As a result, garlic products have been developed, aiming mainly to minimize unpleasant odors and improve their functional properties. However, while heating reduces the strong flavor, it also decreases the antioxidant potential (113). Blanching, roasting, frying, high-pressure processing, and treatment with various chemical additions are known processes that can alter the biochemical process and delay the onset of quality losses. Processed garlic contains a wider variety of organosulfur compounds than those present in the raw garlic clove because allicin is thought to be a transitory component that breaks down rapidly into other sulfur-containing compounds (86). Although allicin is unstable, it rapidly degrades to volatile organosulfur compounds, including diallyl sulfide, diallyl disulfide, diallyl trisulfide, and allyl methyl disulfide, which are responsible for garlic’s distinctive pungent odor (114). To conclude, during food production, storage, and processing, the chemical components found in processed garlic were chemically altered (115). The following are major garlic products available widely in markets.

### 5.1. Garlic oil

Essential oils are plant extracts frequently applied in medicine, food, and other industries for their ability to enhance human and animal nutrition and antibiotic, antibacterial, and antioxidant properties (116). Sulfur-containing compounds are the major component of essential garlic oil, which can be extracted from fresh garlic cloves (117). Diallyl sulfide, diallyl disulfide, and triallyl trisulfide are the three primary biologically active components in garlic oil (118). These chemical compounds can prevent bacterial infections, slow the aging process, regulate the immune system, and act as an anti-inflammatory medications (119, 120). Garlic oil has also been demonstrated to assist people in weight loss programs by lowering LDL cholesterol levels (121). Steam distillation and solvent extraction are commonly used to produce garlic oil (122). To obtain the essential oil, whole garlic cloves were crushed in water and then distilled using heat or extracted with an organic solvent (123). Over 20 sulfides have been reported in steam-distilled garlic oil and oil-soluble extract, with many containing an allyl group responsible for the unique smell and flavor after consuming garlic (17). The chemical composition of these sulfides varies with the temperature and duration of extraction (124). Table 4 shows the extraction method used in garlic oil extraction. The employed extraction technique had an impact on the extraction’s duration and yield. Commercial garlic oils are extensively diluted with other vegetable oils due to their high sulfur content (104).

**Table 4:** Different garlic oil recovery approaches.

Extracting technique	Condition			Chemical properties		Reference
	Duration	Solvent	Temperature	Yield	Alliin content (mg/ml)	
Hydro distillation	2 h	Water	N/R	0.36%	0.88	Somamno et al. (122)	3 h	Water	100°C	0.31%	N/R	Sowbhagya et al. (125)
Hydro distillation with enzyme pre-treatment	3 h	Water	100°C	0.57%	N/R	Sowbhagya et al. (125)
Steam distillation	3 h	Water	100°C	0.28%	N/R	Sowbhagya et al. (125)
Steam distillation with enzyme pre-treatment	3 h	Water	100°C	0.51%	N/R	Sowbhagya et al. (125)
Solvent extraction	4 h	Hexane	N/R	6%	N/R	Rafe and Nadjafi (126)	1 h	Ethanol	30-45°C	0.48%	N/R	Yang et al. (127)
Supercritical CO_2_ extraction	N/R	CO_2_	50°C	7%	N/R	Rafe and Nadjafi (126)
Microwave extraction (300-400W)	45 min	N/R	N/R	0.22%	0.15	Somamno et al. (122)

N/R is not reported.

### 5.2. Prebiotic polysaccharide

Polysaccharides are macromolecules composed of numerous monosaccharide units connected by an alpha- or beta- glycosidic bond. The chemical and physical properties of polysaccharide depend on a number of characteristics, including the type of monosaccharide, the arrangement of monosaccharide units, the type of glycosidic linkage, the molecular weight of polysaccharide, and the degree of polymerization. Yan et al. (128) and Zhao et al. (129) revealed that garlic polysaccharides had potential therapeutic effectiveness against a variety of ailments, including diabetes, inflammation, cancer, and infectious disorders. Fructans are non-digestible fibers composed of fructose monomers found in a wide variety of carbohydrate-storing plants, including bananas, onions, garlic, wheat, barley, asparagus, and Jerusalem artichokes (130–132). Fructans with low degrees of polymerization (less than 10) are referred to as oligofructose or fructooligosaccharides (FOS), whereas compounds with high degrees of polymerization (greater than 10) are referred to as inulin (133, 134). They contain prebiotic characteristics, providing numerous health benefits, including increased good gut microbes, improved mineral absorption, decreased diarrhea, and lowered obesity and diabetes (133). Due to its widespread use in food, nutritional supplements, pharmaceuticals, and feed, the global inulin market is estimated to reach 350 million USD in 2024 (135). Currently, most commercial inulin is derived from chicory roots and Jerusalem artichokes, which contain up to 80% of inulin (136, 137). Hot water diffusion is inulin’s primary industrial extraction method (138). On the other hand, garlic is composed of a complex combination of fructose (85%), glucose (14%) and galactose (1%) (139). Solvent extraction, pressured liquid extraction, enzyme-assisted, microwave-assisted, pulse electric field, and ultrasound-assisted extractions are frequently used to extract polysaccharides from garlic (140). Due to their extensive variety of bioactivity and low side effects on human health, polysaccharides have been widely employed as medicines and dietary supplements. Bioactivities associated with garlic polysaccharides include antioxidant activity, antibacterial activity, Immunomodulator activity, and anticancer potential (141). It is apparent that garlic polysaccharides offer numerous health benefits for humans.

### 5.3. Aged garlic extract

The aged garlic extract was produced by extracting sliced garlic in aqueous or ethanol and aging it naturally for up to 20 months, after which the unpleasant and irritating components in garlic were naturally transformed into more stable and more efficient compounds (142). Table 5 illustrates the solvent and conditions used in the aging process. SAC and trans-S-1-propenyl-L-cysteine are the sulfur-containing amino acids mainly found in alcoholic and aqueous garlic extracts, while allicin, vinyldithiins, ajoene, and diallyl disulfide (DADS) diminished (147, 148). As mentioned, SAC is one of the key sulfur-containing amino acid molecules probably responsible for garlic’s therapeutic properties, which include antioxidant, anticancer, antihepatotoxic, and neurotrophic properties (147). Furthermore, these sulfur-containing compounds contribute to their antioxidant, anti-hypertensive, and immunomodulatory properties, protecting against oxidation, free radicals, cancer, and cardiovascular diseases (149). Garlic extract also possesses antimicrobial properties and is effective against oral bacterial species, particularly gramme-negative bacteria (64). The aging process influences the aroma profile of the product. Therefore, the intense odor of fresh garlic reduces after aging because the thiosulfinates and sulfides responsible for fresh garlic’s pungent odor are thought to decay and be altered to other compounds (150).

**Table 5:** Different approaches for aging garlic and their physiochemical properties.

Aging technique	Condition		Physiochemical properties		Reference
	Concentration	Duration	Polyphenol	SAC content	
Ethanol aging	20%	20 months	N/R	0.2 %	Morihara et al. (143)	15%	20 months	0.07%	N/R	Nencini et al. (144)	N/R	10 months	N/R	0.21%	Imai et al. (145)
Aqueous aging	Water	20 days	N/R	0.18%	Wang et al. (146)

N/R is not reported.

### 5.4. Black garlic

Black garlic processing entails heating the raw material at a high temperature and saturated humidity for a month without adding additives until the flesh turns black by the Maillard reaction and caramelization (151). As a result, its texture becomes more elastic, with a sweet–sour flavor and a milder odor (152). In this process, reducing sugars, amino acids, and antioxidative chemicals increased, making it an interesting functional food product recognized globally (151). The functional ingredients in black garlic, in particular, possess antibacterial, antibiotic, antifungal, antiviral, anticancer, and antioxidant properties (46). Increased SAC content is another significant change that occurs throughout the process. Black garlic has been found to have six times the amount of SAC compared to fresh garlic (153). Nonetheless, the quality of black garlic was influenced by numerous factors. The contents of chemical qualities were exhibited to be genotype-independent, while the processing improved black garlic’s phenolic, flavonoid, and antioxidant properties (9). However, raw material moisture content and processing schemes appeared to directly impact black garlic quality (154, 155). In addition, Zhang et al. (156) and Bae et al. (152) presented that the temperature and duration of processing also directly affected the qualities of black garlic. Table 6 shows the chemical changes in black garlic due to different processing methods.

**Table 6:** The black garlic processing condition and the alteration of chemical component.

Black garlic condition				Chemical properties				Reference
Equipment	Temperature (°C)	Relative humidity (%)	Duration (days)	SAC content	Antioxidant activity	TPC	TFC	
Thermo-hydrostat chamber	40	70	45	↑ 535%	↑ 262%	N/R	N/R	Bae et al. (152)
	55	70	45	↑ 478%	↑ 389%	N/R	N/R	
	70	70	45	↑ 478%	↑ 446%	N/R	N/R	
	85	70	45	↑ 336%	↑ 621%	N/R	N/R	
Rice cooker	75	80	15	N/R	↑ 583%	↑ 2,436%	↑ 185%	Sunanta et al. (9)
Thermohygrostatic chamber	70	90	21	N/R	N/R	↑ 319%	↑ 377%	Choi et al. (46)
Microwave heating	75	80	5	N/R	↑ 338%	↑ 401%	↑ 1,725%	Sunanta et al. (154)

Chemical properties values were compared to those of fresh garlic. ↓ and ↑ indicate a decrease and an increase, respectively, from freshly harvested garlic.

N/R is not reported.

### 5.5. Garlic powder

Garlic powder has a high marketability due to its numerous advantages; for example, it is appealing to consumers because it eliminates the requirement to peel and chop, making it simpler to use in recipes (157). It has an extended shelf life, concentrated flavor, and flexibility, enabling precise flavor control and incorporation in a variety of dishes (158, 159). Its suitability for export and distribution contributes to its market demand. Its lightweight form makes it suitable for export and shipping on a large scale (160). In addition, garlic powder can be compressed into tablets designed to deliver the potential health benefits of garlic in a convenient and consistent dose. It is commonly used as a dietary supplement to promote cardiovascular health, immune function, and overall health (161). A wide range of bioactive substances contribute to the functional properties of garlic powder, which is derived from dehydrated garlic cloves. The bioavailability of these compounds in garlic powder is affected by factors such as processing methods and storage conditions (159, 162). The presence of bioactive compounds, such as allicin derivatives and sulfur compounds, which generate antioxidant effects by scavenging free radicals and protecting against oxidative stress, is responsible for these functional properties. The potential cardiovascular benefits of garlic powder include cholesterol-lowering effects, inhibition of platelet aggregation, and enhancement of blood circulation. Allicin is extremely sensitive and immediately degrades into other sulfur compounds during processing and storage (96). However, some production procedure such as freeze-drying are able to maintain the allicin content of garlic powder (163, 164). In contrast, sulfur compounds that exhibit higher stability, such as diallyl sulfide (DAS), diallyl disulfide (DADS), and diallyl trisulfide (DATS), are more effectively maintained in garlic powder.

## 6. Conclusion

Along with a broad list of the culinary and pharmacological benefits, garlic is one of the most valuable crops in the world. It contains variable of bioactive compounds, the majority of which are sulfur-containing and contribute to the distinctive pungent odor. With its diverse range of bioactive compounds, predominantly sulfur-containing, it imparts the characteristic pungent aroma. This article serves as a valuable resource, offering practical insights into the application and utility of garlic products. Initial processing can extend the storage life of garlic, while various food processing techniques available today can improve the marketability of this highly perishable crop. Curing is the simplest step for extending the shelf life by eliminating excess moisture. However, the curing temperature was found to be the most influential factor on the bioavailability of the ingredients in garlic. The minimal preparation (such as peeling, slicing, and chopping) is intended to increase customer convenience. This approach, however, reduces its shelf life and diminishes its flavor due to tissue damage. Various garlic products, including garlic oil, aged garlic, black garlic, and garlic powder, serve as functional foods. These products aim to mitigate the strong flavors associated with raw garlic while enhancing their functionality and practically for convenience. During processing, garlic undergoes significant changes in its chemical composition. Sulfur-containing compounds are reduced, bioactive compounds increase, and proteins and sugars undergo alterations due to heat treatment. Overall, this article serves as a valuable resource for industry professionals, researchers, and consumers seeking to comprehend the effects of food processing on the functional properties of garlic. It also supports quality control measures and promotes consumer education regarding the effects of garlic processing.

## Author contributions

PiS, VK, TP, KJ, PR, PhS, and SS wrote and reviewed the manuscript. All authors approved the final manuscript.

## Funding

This research project is supported by The National ResearchCouncil of Thailand (NRCT), contact number N41A640335. This research is supported by a grant from Targeted Research, Chiang Mai University and partially supported by Chiang Mai University.

## Conflict of interest

The authors declare that the research was conducted in the absence of any commercial or financial relationships that could be construed as a potential conflict of interest.

## Publisher’s note

All claims expressed in this article are solely those of the authors and do not necessarily represent those of their affiliated organizations, or those of the publisher, the editors and the reviewers. Any product that may be evaluated in this article, or claim that may be made by its manufacturer, is not guaranteed or endorsed by the publisher.
